# Factors affecting the incidence of surgical site infection after geriatric hip fracture surgery: a retrospective multicenter study

**DOI:** 10.1186/s13018-019-1449-6

**Published:** 2019-11-21

**Authors:** Xiaopo Liu, Zhijie Dong, Jun Li, Yunbo Feng, Guolong Cao, Xin Song, Jie Yang

**Affiliations:** 1 Third Department of Orthopaedics, Tangshan Gongren Hospital, Tangshan, Hebei 063000 People’s Republic of China; 2 Department of Orthopaedic Surgery, Hebei General Hospital, Shijiazhuang, Hebei 063000 People’s Republic of China; 3 Second Department of Orthopaedic Surgery, First Hospital of Hebei Medical University, Shijiazhuang, Hebei 063000 People’s Republic of China; 4 Department of Radiology, Tangshan Gongren Hospital, Tangshan, Hebei 063000 People’s Republic of China; 5 First Department of Geriatric, Tangshan Gongren Hospital, Tangshan, Hebei 063000 People’s Republic of China

**Keywords:** Geriatric patients, Hip fracture, Risk factors, Serum albumin, SSI

## Abstract

**Background:**

Geriatric hip fracture is a common type of osteoporotic fracture with high mortality and disability; surgical site infection (SSI) can be a devastating complication of this injury. By far, only a few studies identified easily remediable factors to reduce infection rates following hip fracture and less researches have focused on geriatric patients. The objective of this study was to identify potentially modifiable factors associated with SSI following geriatric hip fracture surgery.

**Methods:**

This retrospective, multicenter study involves three level I hospitals. A total of 1240 patients (60 years or older) underwent hip surgery with complete data were recruited between January 2016 and June 2018. Demographics information, medications and additional comorbidities, operation-related variables, and laboratory indexes were extracted and analyzed. Receiver operating characteristic (ROC) analysis was performed to detect the optimum cut-off value for quantitative data. Univariate and multivariate logistic analysis model were performed respectively to identify the independent predictors.

**Results:**

Ninety-four (7.58%) patients developed SSI in this study, and 76 (6.13%) had superficial infection, while 18 (1.45%) were diagnosed with deep infection. Results of univariate and multivariate analysis showed age > 79 years (OR, 2.60; *p* < 0.001), BMI > 26.6 kg/m^2^ (OR, 2.97; *p* < 0.001), operating time > 107 min (OR, 2.18; *p* = 0.001), and ALB < 41.6 g/L (OR, 2.01; *p* = 0.005) were associated with an increased incidence of SSI; drainage use (OR, 0.57; *p* = 0.007) could reduce the incidence of wound infection for patients after geriatric hip fracture.

**Conclusion:**

Accurate modifiable variables, operating time > 107 min, serum albumin < 41.6 g/L, BMI > 26.6 kg/m^2^, and age > 79 years could be applied to distinguish geriatric patients with high-risk of postoperative surgical site infection.

Hip fracture is a common type of osteoporotic fracture, with high mortality and disability. Previous study, in the UK, has reported that there are more than 86,000 hip fractures annually [1], and in the next 40 to 50 years, more than 7 million patients with hip fractures are anticipated worldwide each year [2], and more than 50% of all osteoporotic hip fractures will occur in Asia owing to the growing population size [3]. With the austere circumstance of aging population all over the word, geriatric hip fracture has gradually become an important challenge for health-care systems and orthopedists in every country.

A large proportion of the mortality and cost associated with hip fractures can be attributed to postoperative complications, of which surgical site infection (SSI) is a major cause. One-year mortality for patients with SSI was 35.4–50.0%, which was substantially higher than those without wound infection (24.1–30%) [4, 5]. From an economic perspective, SSI accounts for 17% of nosocomial infections and cost between 1 and 10 billion US dollars annually [6]. Risk factors associated with wound infection which were established in previous studies included body mass index (BMI) [7], smoking [8], urinary tract infection [9], female gender [10], delay in surgery (more than 1 week) [11], prolonged operating time [12], and surgical type (hemiarthroplasty) [12]. However, most of these predictors were unmodifiable, and the findings might be partially compromised by the limited sample size of these studies; moreover, some of these investigations possess the feature of single center. By far, only a few studies identified easily remediable factors to reduce infection rates following hip fracture and less researches have focused on geriatric patients.

The object of this multicenter study was to identify potentially modifiable factors associated with surgical site infection following geriatric hip fracture surgery.

## Patients and methods

This retrospective, multicenter study involving three level I hospitals in Hebei Province of China. We evaluated the data of all patients admitted to the orthopedic department with hip fracture from January 2016 to June 2018. During the time window, all patients aged 60 or older who underwent osteosynthesis or hemi- and total hip arthroplasty due to traumatic injuries were retrospectively enrolled and relevant data were collected.

The exclusion criteria were (1) periprosthetic fractures, (2) old fractures (> 21 days from trauma), (3) tuberculosis of hip joint, (4) pathological fracture (primary or metastatic tumor), (5) hip fracture treatment by conservative methods (skin or skeletal traction; plaster immobilization or thermoplastic plate brace), (6) open fractures, and (7) patients with incomplete data of medical record or follow-ups after discharge. All the patients were followed up for any evidence of SSI occurrence, via telephone assessment and collection of medical records postoperatively.

According to the published literatures and our study hypothesis, we selected approximately 60 variables that could be associated with the occurrence and development of a surgical site infection after geriatric hip fractures, such as gender, age, body mass index (BMI, underweight, < 18.5; normal, 18.5–23.9; overweight, 24–27.9; obesity, 28–31.9; morbid obesity, 32 and more), residential status, occupation, medications and additional comorbidities (hypertension, rheumatoid disease, diabetes mellitus, liver and kidney function, anemia, benign or malignant tumor, respiratory disorders, immune system disease, cardio or cerebrovascular disease, allergic history), previous surgery for a fracture of the hip, and other musculoskeletal site.

Perioperative factors were also extracted from the electronic medical records (EMRs) by our well-trained investigators. These variables including time to surgery (days between admission and operation), season of surgery (summer or autumn), level of surgeon (attending, associate chief, chief), surgery type (emergent or elective), anesthesia time, anesthetic type (intrathecal, general, combination), operating time, intraoperative body temperature, intraoperative blood loss and transfusion (ml), use of antibiotics, drainage usage, length of stay in hospital, and American Society of Anaesthesiologists (ASA, I-V) score [13] were performed to evaluate the patients’ comorbidities and physical status.

In order to assess the general condition of the geriatric patients, we recorded the preoperative laboratory indexes (24 h within hospitalization) into analysis. The biochemical indicator includes potassium (K^+^), sodium (Na^+^), chloride ion (Cl^+^), calcium (Ca^+^), phosphonium (P^+^), magnesium (Mg^+^), serum total protein (TP), albumin (ALB), globulin (GLOB), and A/G value (albumin/globulin); routine hematology indexes includes white blood cell (WBC), neutrophil granulocyte (NEUT), lymphocyte (LYM), monocyte (MON), eosinophil granulocyte (EOS), basophilic granulocyte (BAS), red blood cell (RBC), hemoglobin (HG), blood platelet (PLT), and blood glucose (GLU); infection contagion indexes includes hepatitis B virus antigen (anti HBV), hepatitis C virus antigen (anti HCV), and human immunodeficiency virus antigen (Anti HIV).

The definition of surgical site infection was based on the criteria of the United States Center for Disease Control and Prevention [14]. This classification system categorizes SSI into three types: (1) superficial incisional, (2) deep soft tissue of the incision, and (3) organ/space infection. Superficial SSI, defined as infection occurring within 30 days postoperative, involves the skin and subcutaneous tissue of the surgical site only; one or more symptom is observed: redness, swelling, pain of the incision, purulent discharge, spontaneous wound dehiscence, and positive results of bacterial culture. Deep wound infection is defined as infection that occurs within 90 days post-operation, and involves the fascial and muscular layer. SSI refers to bone or hip joint diagnosed with organ/space infection, according to the previous study [9].

This study was approved by the Ethics Committee of all the participant hospitals and received written consent from all the study participants.

## Statistical analysis

**Fig. 1:**
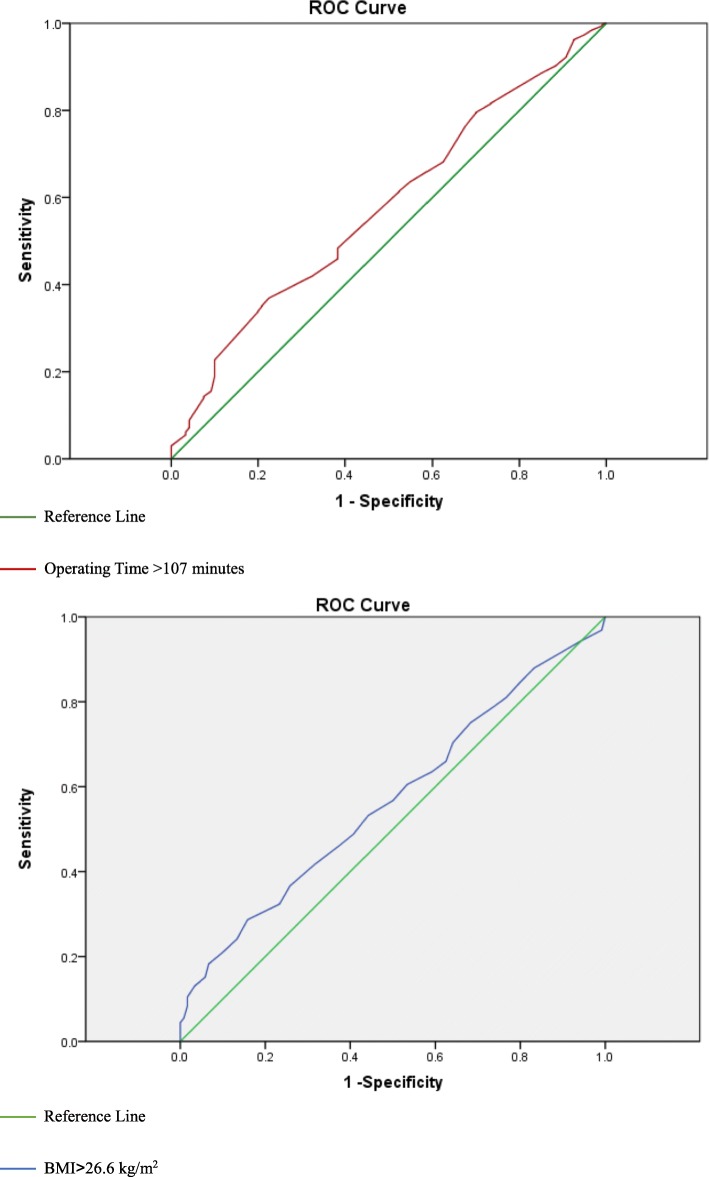
A receiver operating characteristic (ROC) curve analysis used to evaluate the optimum cut-off value of the continuous variable

Receiver operating characteristic (ROC) analysis was performed to detect the optimum cut-off value for quantitative data, such as length of stay in hospital, BMI, intraoperative blood loss and temperature, operating time, anesthesia time, WBC, EOS, RBC, HGB, PLT, ALB, GLOB, and other laboratory indexes. Univariate logistic analysis was used to assess the correlation between categorical variable and SSI. Whitney *U* test was used for non-normally distributed continuous variables and *t* test for normally distributed variables. Factors, demonstrated increasing the risk of SSI in univariate model (*p* < 0.1), entered into the multivariable logistic regression analysis to identify independent predictors of wound infection. Hosmer–Lemeshow test was used to evaluate goodness-of-fit of this model, and an acceptable fitness was enacted as *p* > 0.05. In univariate and multivariable analysis, a *P* value < 0.05 was considered to be statistically significant. All the tests were performed using the SPSS 19.0 software package (SPSS Inc., Chicago, Illinois) (Fig. 1).

## Results

During the follow-up period (24–41 months, mean 25.8 ± 8.6 months), a total of 1240 patients (mean age 75.8 ± 8.9 years) from three hospitals were enrolled in this study; of them, 325 (26.2%) were male (mean age 73.9 ± 7.9 years) and 915 (73.8%) were female (mean age 76.5 ± 9.2 years), with a male-female ratio of 1:2.8. Further, 553 (44.6%) patients underwent open or close reduction and internal fixation; 294 (23.7%) patients underwent hemiarthroplasty and total hip arthroplasty in 393 (31.7%) patients. Surgery was performed at the mean 4.2 days (range 1–14 days), and the mean operating duration was 86.3 ± 30.5 min.

Among the 1240 patients, a total of 94 SSIs were observed, demonstrating an overall incidence rate of 7.58%. These 94 patients were specifically classified as superficial incision infection in 76 case and deep infection in 18 patients, giving the incidence of 6.13% and 1.45% for superficial and deep SSI respectively. The mean length of hospitalization for SSIs and no-infection groups were 18.8 days vs. 13.3 days, and the difference was statistically significant (*p* = 0.010). The earliest diagnosis of SSI was 4 days postoperatively and latest at 113 days after hospital discharge. LYM and ALB in SSI group were 1.28 × 10^9^/L and 38.50 g/L; these two indexes were both lower than that in non-SSI group (1.62 × 10^9^/L and 40.37 g/L), and there is significant difference for variables mentioned above between two groups (*p* < 0.001). Microorganism examination and drug sensitivity results showed that *Staphylococcus aureus* was the most common causative agent, followed by *Pseudomonas aeruginosa*, *Enterococcus faecalis*, Methicillin-resistant *S*. *aureus* (MRSA), *Staphylococcus epidermidis*, and *Enterobacter cloacae*.

Receiver operating characteristic (ROC) analysis was performed to detect the optimum cut-off value for continuous variables which could be associated with SSI. According to the criteria of ROC, the test accuracy is low when area under the cure (AUC) ranges from 0.5 to 0.7; the variables demonstrated to possess a moderate accuracy when AUC between 0.7 and 0.9, and the accuracy is satisfactory high when the AUC > 0.9. Table 1 presented the AUC and their corresponding 95% confidence interval and the optimum cut-off value for these quantitative data. From this we could see that AUC of these continuous variables ranges from 0.555 to 0.676 which indicates a low accuracy.

**Table 1:** Continuous variables tested by receiver operating characteristic (ROC) analysis

Variables	Area under the cure (AUC)	95% CI	Optimum cut-off value
Basophilic granulocyte (BAS, 10^9^/L)	0.676	0.621–0.731	0.05
Lymphocyte (LYM, 10^9^/L)	0.555	0.502–0.609	1.26
Operating time (min)	0.578	0.526–0.631	107
Body mass index (BMI, kg/m^2^)	0.555	0.508–0.602	26.6
Age (year)	0.568	0.517–0.620	79
Serum albumin (ALB, g/L)	0.560	0.508–0.612	41.6
Anesthesia time (min)	0.576	0.519–0.633	147

The results of univariate analysis are shown in Table 2. Among the interesting variables, ten factors were confirmed affecting the incidence of surgical site infection after geriatric hip-fracture: age > 79 years (OR = 2.15, 95% CI = 1.29–3.60), residence of rural area (OR = 1.80, 95% CI = 1.11–2.90), BMI > 26.6 kg/m^2^ (OR = 3.45, 95% CI = 1.94–6.15), operating time > 107 min (OR = 2.01, 95% CI = 1.28–3.17), ALB < 41.6 g/L (OR = 1.93, 95% CI = 1.21–3.10), LYM < 1.26 × 10^9^/L (OR = 1.58, 95% CI = 1.08–2.33), intraoperative and postoperative intravenous of antibiotics (OR = 0.41, 95% CI = 0.21–0.80; OR = 0.25, 95% CI = 0.11–0.59), drainage use (OR = 0.62, 95% CI = 0.42–0.91), and ASA classification ≥ III (OR = 2.15, 95% CI = 1.87–4.59). All of the ten above-mentioned prognostic factors were entered into multivariable logistic regression model to verify the independent predictors of SSI. Final statistical results demonstrated, age > 79 years (OR = 2.60; 95% CI = 1.53–4.43; *p* < 0.001), BMI > 26.6 kg/m^2^ (OR = 2.97; 95% CI = 1.65–5.36; *p* < 0.001), operating time > 107 min (OR = 2.18; 95% CI = 1.35–3.51; *p* = 0.001), and ALB < 41.6 g/L (OR = 2.01; 95% CI = 1.23–3.29; *p* = 0.005) were associated with an increased incidence of SSI; moreover, the use of drainage (OR = 0.57; 95% CI = 0.37–0.86; *p* = 0.007) could reduce the incidence of wound infection following geriatric hip-fracture (Table 3). The result of Hosmer–Lemeshow test demonstrated a preferable fitness (*X*^2^ = 5.859, *p* = 0.663).

**Table 2:** Results of univariate analysis to detect association between factors studied and SSI

Variables	SSIs (*n* = 94, 7.58%)	Non-infection group (*n* = 1146, 92.42%)	*P* value
Age > 79	18 (19.1)	419 (36.6)	0.004^a^
Surgery type			0.764
Emergency surgery	93 (98.9)	1131 (98.7)	
Selective surgery	1 (1.1)	15 (1.3)	
Season of surgery (Summer)	45 (47.9)	741 (64.7)	0.947
Gender (Male)	40 (42.6)	285 (24.9)	0.052
Place of residence			0.017^a^
Rural area	74 (78.7)	607 (53.0)	
Urban area	20 (21.3)	539 (47.0)	
BMI (> 26.6)	81 (86.2)	580 (50.6)	0.000^a^
Diabetes mellitus	20 (21.3)	151 (13.2)	0.575
Hypertension	46 (48.9)	467 (40.8)	0.927
Anemia	3 (3.2)	23 (2.0)	0.771
Smoking	16 (17.0)	106 (9.2)	0.161
Liver disease	2 (2.1)	17 (1.5)	0.700
ASA score			
III	46 (48.9)	566 (49.4)	0.027^a^
IV	2 (2.1)	29 (2.5)	
Intraoperative bleeding (ml)			
≤ 400	78 (83.0)	862 (75.2)	0.504
> 400	16 (17.0)	281 (24.5)	
Operating time (> 107 min)	22 (23.4)	373 (32.5)	0.002^a^
Intraoperative body temperature			0.300
< 36.3	25 (26.6)	131 (11.4)	
≥ 37.3	2 (2.1)	23 (2.0)	
Intraoperative antibiotics use	87 (92.6)	826 (72.1)	0.009^a^
Postoperative antibiotics use	90 (95.7)	724 (63.2)	0.001^a^
TP^**b**^ (< 58 g/L)	13 (13.8)	111 (9.7)	0.767
ALB^b^ (< 41.6 g/L)	74 (78.7)	596 (52.0)	0.006^a^
GLOB^b^ (< 20 g/L)	6 (6.4)	68 (5.9)	0.224
GLU^b^ (> 6.10 mmol/L)	47 (50.0)	472 (41.2)	0.593
WBC^b^ (10^9^/L)			0.421
References [4–10]	74 (78.7)	894 (78.0)	
< 4	0 (0.0)	82 (1146)	
> 10	20 (21.3)	170 (14.8)	
LYM^b^ (< 1.26^a^10^9^/L)	55 (58.5)	367 (32.0)	0.020^a^
MON^b^ (10^9^/L)			0.471
References (0.1–0.6)	61 (64.9)	805 (70.2)	
< 0.1	4 (4.3)	5 (0.4)	
> 0.6	29 (30.9)	336 (29.3)	
EOS^b^ (10^9^/L)			0.061
References (0.02–0.52)	71 (75.5)	947 (82.6)	
< 0.02	23 (24.5)	176 (15.4)	
> 0.52	0 (0.0)	23 (2.0)	
BAS^b^ (< 0.05^a^10^9^/L)	89 (94.7)	978 (85.3)	0.530
PLT^b^ (10^9^/L)			0.844
References(100–300)	82 (87.2)	1017 (88.7)	
< 100	3 (3.2)	17 (1.5)	
> 300	9 (9.6)	112 (9.8)	
Drainage use	51 (54.3)	387 (33.8)	0.015^a^

^a^Significant variables

^b^*TP* total protein, *ALB* albumin, *GLOB* globulin, *GLU* blood glucose, *WBC* white blood cell, *NEUT* neutrophil, *LYM* lymphocyte, *MON* monocyte, *EOS* eosinophils, *BAS* basophilic, *PLT* blood platelet count

**Table 3:** Multivariable logistic regression analysis of factors associated with SSI after geriatric hip fracture

Variable	*P* value	Odds ratio	95% CI
Age > 79 years	0.000^a^	2.60	1.53–4.43
BMI > 26.6 kg/m^2^	0.000^a^	2.97	1.65–5.36
Operating time > 107 min	0.001^a^	2.18	1.35–3.51
ASA ≥ III	0.179	2.26	0.69–7.41
ALB < 41.6 g/L	0.005^a^	2.01	1.23–3.29
Drainage use^b^	0.007^a^	0.57	0.37–0.86

^a^Significant variables

^b^Protective factor

## Discussion

Surgical site infection (SSI) after hip surgery is a devastating complication for patients, especially for the elderly. In recent years, the incidence of hip fracture is on the rise, attracting more and more attention of the orthopedic surgeons. It has been reported that the 1-year mortality of geriatric patients with hip fracture is 26~ 29%, and the 2-year mortality is 38% [15–17], which seriously threatens the quality of life and health for geriatric people. In the present study, the overall incidence of SSI was 7.58%, with 6.13% and 1.45% for superficial and deep SSI respectively. Our main findings were that five potentially modifiable factors (four risk and one protective) were significantly associated with occurrence of wound infection, namely age > 79 years, BMI > 26.6 kg/m^2^, operating time > 107 min, ALB < 41.6 g/L, and postoperative use of drainage.

It has been previously reported that incidence of SSI following hip fractures ranges from 2 to 9.5% [5, 8, 18, 19], and deep SSI rate was 1.2–3.6% [4, 5, 20]. Overall incidence of wound infection in our study was 7.58%; this rate was consistent but relatively higher than that in other studies which focused on geriatric hip fracture. In the present investigation, we recruited 1240 patient who were admitted to the orthopedic department of three different level I hospitals. The special health-care systems including transfer (dual referral), and medical insurance systems in China make injury severity of patients in different-level hospitals hugely different, and elderly patients with complex additional comorbidities and intractable physical condition were more likely to underwent surgery in the level I hospital. The mean age of patients studied is 75.9 ± 9.0 years in this study, and 254 (20.5%) patients suffered from hip fracture due to high energy trauma; hypertension was diagnosed in 573 (46.2%) patients, benign and malignant tumor in 11 (0.9%) patients, coronary heart disease in 252 (20.3%) patients, and diabetes mellitus in 216 (17.4%) cases respectively. Given these circumstances, SSI incidence rate may increase accordingly. Mean length of stay in hospital for infected patients was 23.0 days, and this value increased to 28.3 days in patients who suffered from deep infection. SSI prolonged 6.3 days of hospitalization compared to the non-infected patients (mean 16.7 days, SD 8.2 days), and this difference is statistically significant (*p* = 0.032). Infection rates after internal fixation ranges from 1 to 30%, which is higher as compared to elective joint replacement. It has been confirmed that periprosthetic joint infection occurs in 1–2% of primary arthroplasties and 4% of revision arthroplasties [21]. SSI generally occurs exogenously due to the trauma itself, during insertion of the fixation device, or during disturbed wound healing [22]. *Staphylococcus aureus* and MRSA were the two most common microorganisms cultured in SSIs after hip fracture in this present study, and the result was in accordance with those previous published articles [23].

Age has been widely confirmed as an independent risk factors for wound infection after operation following hip and other fractures [24–27]. Age > 79 years was significantly associated with SSI (*p*, 0.000) and the incidence of SSI in patients aged older than 79 years would increase to more than two times (OR, 2.60) compared to the non-infected people. It is known that people with an advanced age often suffered from concomitant comorbidities, such as hypertension, coronary heart and kidney disease. Advanced age is prone to a higher incidence of SSI; one explanation for this is that the presence of comorbidities will often cause an individual to have a much lower baseline exercise tolerance compared with those who are systemically well.

As a vital indicator in orthopedics and other disciplines, operating time affects the incidence of SSI in many ways. A longer operation duration may attribute to difficulty of the surgical procedure and this condition may lead to extensive tissue stripping, prolonged exposure of incision to some potential infectious factors. de Jong L et al. [10] applied 45 min and 90 min (the 15th and 85th percentile) as cut-off value based on the previous study [28, 29], and they found that operating time longer than 90 min increased the risk of SSI to 2.66 times in patients after hemiarthroplasty following femoral neck fracture. Similar conclusion was demonstrated in Capdevila A’ investigation [30]; the author retrospectively reviewed 657 patients who underwent hip fracture surgery from 2012 to 2013, and statistical results showed that length of surgery longer than 120 min was independently associated with SSI (OR, 4.5). However, these previous studied failed to demonstrate a definite and highly sensitive cut-off value for operating time to identify patients with high risk of postoperative wound infection. We innovatively advocated that surgical duration longer than 107 min would significantly increase the surgical incision infection rate (OR, 2.18; *p*, 0.001). We surgeons have designed and implemented numerous studies, with the aim of reducing the incidence of postoperative infection, to describe the association between clinical factors and SSI. Based on our important founding, more patients who are at risk of SSI potentially can be identified with the new cut-off point of (operating time > 107 min) compared to 120 min and other value. According to the results, when the operating time comes to 120 min, incidence of infection would increase to 2.64 times, with a *P* value and 95% CI of 0.002 and 1.42–4.90 respectively in our study. Therefore, we think operation duration > 107 min is a more remarkable prognostic factor for the occurrence of SSI following geriatric hip fracture surgery.

Nutritional deficiency has also been confirmed to be an independent risk factor of postoperative complications, including infection, prolonged length of stay, hematoma, and so on. Serum albumin < 35 g/L is defined as malnutrition in previous studies, and this criterion is widely accepted by researchers. Oztürk et al. [31] demonstrated in their article that there were 55.4% geriatric patients that were malnourished following hip fractures. Pimlott et al. evaluated 583 patients with hip fractures and found that levels of albumin were abnormal in 55% [32]. However, in the present study, malnourish rate for elderly patients was 15.8% which indicated a better state of nutrition for our study sample. ABL was grouped to normal when the level ranges from 40.0 to 55.0 g/L. Inconsistent with previous founding, we identified that ALB lower than 41.6 g/L instead of 35 g/L was associated with postoperative SSI, and ALB < 35 g/L was not a risk factor of SSI in univariate analysis (*p* = 0.527, OR = 1.18, 95% CI 0.71–1.94) in the present study. Based on our accurate standard of ALB level, more sub-high-risk geriatric patients could be distinguished from suffering from wound infection.

Body mass index (BMI) was calculated as weight divided by the square of height, and was grouped according to the Chinese reference criteria: overweight, 24–27.9; obesity, 28–31.9; morbid obesity, ≥ 32. Patients with a higher BMI were more likely to encounter postoperative wound complications such as SSI and delayed healing compared to normal ones. Ma T et al. [8] retrospectively investigated 611 patients age 65 or older who underwent surgery for hip fracture and they found that every extra 1 kg/m^2^ of BMI increased the risk of SSI approximately by 12%. However, the relationship between BMI and SSI has remained constant in some initial investigations. According to the results of Akinleye’s [7] study, a low weight (BMI < 20) and morbidly obese (BMI > 40) patients had both the highest mortality rates and the lowest superficial infection rates, but deep SSI increased linearly with increasing BMI. Our study clarified the role that BMI plays during the postoperative course and confirmed that BMI > 26.6 kg/m^2^ was an independent predictor of wound infection. Similarly, obesity and morbid obesity (BMI > 28; *p* = 0.000, OR = 4.56, 95% CI 2.04–9.6) significantly increased the risk of SSI for Chinese elderly patients undergoing surgical intervention after hip fracture in this study. We also observed an interesting phenomenon: malnutrition rate of patients with a BMI lower than 26.6 was 15.5%, and 16.5% for the rest of the patients (BMI > 26.6); this finding demonstrated an uncorrelated relationship between BMI and level of ALB in predicting risk of SSI.

The present study also investigated the relationship between drainage use and SSI. Univariate and multivariable logistic regression model (odds ratio = 0.361; 95% CI = 0.137–0.953) demonstrated that drainage use was an independent protective factor of postoperative wound healing (OR = 0.61, 95% CI 0.42–0.91; OR = 0.57, 95% CI 0.37–0.86). However, practices of drainage system remained controversial in previous studies. Some authors advocated that wound drainage has no benefit in joint arthroplasty [33–35], and a shorter wound drainage time was associated with the prevention of postoperative SSI. Collection of fluids in surgical incision provides pabulum for bacteria growth, and impairs wound healing by increasing incision tension and reducing tissue perfusion [36]. In our study, the infection rate in patients with drainage use is much lower than that without drainage systems (5.4% versus 11.6%).

Although the ASA classification [37], delayed in surgery and duration of anesthesia, are good predictors of SSI, the findings of the present investigation do not conclusively associate these variables with postoperative wound infection.

The present study had several strengths; it evaluated the association between both clinical variables, laboratory indexes, and geriatric hip fracture in a large population. Among these continuous variables, receiver operating characteristic (ROC) analysis was performed to define a highly sensitive cut-off value. A figure of modifiable factors such as operating time > 107 min, serum albumin < 41.6 g/L, and BMI > 26.6 kg/m^2^ were clearly demonstrated by this investigation. However, some particular limitations of our study should be recognized. Firstly, retrospective study inevitably inherits the selective bias; secondly, confounding factors such as postoperative biochemical indexes, and other variables may affect the occurrence of infection.

In summary, overall incidence of SSI for geriatric patients following hip fracture surgery was 7.58%. Modifiable variables, operating time > 107 min, serum albumin < 41.6 g/L, BMI > 26.6 kg/m^2^, and age > 79 years were demonstrated as independent risk factors of postoperative wound infection. Drainage use could affect occurrence of SSI by reducing the risk.

## Data Availability

The datasets generated and/or analyzed during the current study are not publicly but are available from the corresponding author on reasonable request.

## Abbreviations

ALB: Albumin
ASA: American Society of Anesthesiologists
BAS: Basophilic granulocyte
BMI: Body mass index
CDC: United States Center for Disease Control
EMRs: Electronic medical records
GLOB: Globulin
GLU: Blood glucose
HG: Hemoglobin
LYM: Lymphocyte
MON: Monocyte
NEUT: Neutrophil granulocyte
PLT: Blood platelet
RBC: Red blood cell
SSI: Surgical site infection
TP: Serum total protein
WBC: White blood cell
